# PWHATSHAP: efficient haplotyping for future generation sequencing

**DOI:** 10.1186/s12859-016-1170-y

**Published:** 2016-09-22

**Authors:** Andrea Bracciali, Marco Aldinucci, Murray Patterson, Tobias Marschall, Nadia Pisanti, Ivan Merelli, Massimo Torquati

**Affiliations:** 1 Computer Science and Mathematics, School of Natural Sciences, Stirling University, Stirling, FK9 4LA UK; 2 Department of Computer Science, University of Torino, Torino, Italy; 3 Laboratoire de Biométrie et Biologie Evolutive, University Claude Bernard, Lyon, France; 4 Center for Bioinformatics, Saarland University, Saarland, Germany; 5 Max Planck Institute for Informatics, Saarbrücken, Germany; 6 Department of Computer Science, University of Pisa, Pisa, Italy; 7 Erable Team, INRIA, Grenoble, France; 8 Institute of Biomedical Technologies, National Research Council, Milan, Italy

**Keywords:** Haplotyping, High-performance computing, Future generation sequencing

## Abstract

**Background:**

Haplotype phasing is an important problem in the analysis of genomics information. Given a set of DNA fragments of an individual, it consists of determining which one of the possible alleles (alternative forms of a gene) each fragment comes from. Haplotype information is relevant to gene regulation, epigenetics, genome-wide association studies, evolutionary and population studies, and the study of mutations. Haplotyping is currently addressed as an optimisation problem aiming at solutions that minimise, for instance, error correction costs, where costs are a measure of the confidence in the accuracy of the information acquired from DNA sequencing. Solutions have typically an exponential computational complexity. WhatsHap is a recent optimal approach which moves computational complexity from DNA fragment length to fragment overlap, i.e., coverage, and is hence of particular interest when considering sequencing technology’s current trends that are producing longer fragments.

**Results:**

Given the potential relevance of efficient haplotyping in several analysis pipelines, we have designed and engineered pWhatsHap, a parallel, high-performance version of WhatsHap. pWhatsHap is embedded in a toolkit developed in Python and supports genomics datasets in standard file formats. Building on WhatsHap, pWhatsHap exhibits the same complexity exploring a number of possible solutions which is exponential in the coverage of the dataset. The parallel implementation on multi-core architectures allows for a relevant reduction of the execution time for haplotyping, while the provided results enjoy the same high accuracy as that provided by WhatsHap, which increases with coverage.

**Conclusions:**

Due to its structure and management of the large datasets, the parallelisation of WhatsHap posed demanding technical challenges, which have been addressed exploiting a high-level parallel programming framework. The result, pWhatsHap, is a freely available toolkit that improves the efficiency of the analysis of genomics information.

## Background

In diploid individuals, such as humans, each chromosome exists in two copies, also referred to as *haplotypes*. One haplotype is inherited from the father while the other haplotype is inherited from the mother. Although these two copies are highly similar, they are not identical, reflecting the genetic differences between mother and father. A Single Nucleotide Polymorphism (SNP) is a variation of a single nucleotide that occurs at a specific position, called locus, in the pair of sequences. Given a set of heterozygous variants, i.e., loci where the two alleles differ, e.g. SNPs, the problem of assigning each of the two alleles at each locus to one of the two haplotypes is known as *phasing*.

Phasing SNPs is important for many applications. Haplotype-resolved genetic data allows studying epistatic interactions, for instance. Gene regulation and epigenetics have also been demonstrated to be haplotype specific in many instances [1]. One of the prime uses of haplotype panels, i.e., large sets of haplotypes present in a population, lies in the *imputation* of missing variants, which is instrumental for lowering costs and boosting power of genome-wide association studies [2]. Not surprisingly, constructing high-quality haplotype panels for human populations has been one of the central goals of several large-scale projects [3–6]. Further uses of haplotype data include studying evolutionary selection, population structure, loss of heterozygosity, and for determining the parental origin of de novo mutations. Refer to [7] for a detailed review of these and other applications.

Currently, the most prevalent phasing tools use genotype information for a large number of individuals as input. Therefore, phase information has not been observed directly, but is inferred based on the assumption that haplotype tracts are shared between individuals in a population. The resulting approaches are statistical in nature, based on, e.g., latent variable modeling [8–10], and Markov chain Monte Carlo (MCMC) techniques [11].

Noticeably, one of the major drawbacks of these statistical phasing methods is the lack of *direct* information that pairs of neighboring SNPs are on the same haplotype – something that is ultimately needed if one is chaining together the SNPs to form a pair of haplotypes. This can be provided by a sequencing *read*, i.e., a fragment of the actual DNA sequence. The existence of a read containing a pair of heterozygous SNPs is direct evidence that they come from the same haplotype. However, current sequencing technologies often do not provide long enough reads to sufficiently link neighboring SNPs. This is why the most widely used phasing methods are based on statistical information compiled from a large amount of data about the relationship between SNPs, such as linkage disequilibrium [12], or from patterns that arise in existing haplotypes, such as these aforementioned haplotype panels [3].

It is long reads that will really solve this problem, one of the major reasons for the recent interest in long-read technologies. While still not competitive in terms of per-base cost and error rates, and not yet sufficient to completely overcome the above drawbacks, cutting edge technologies such as PacBio’s Single Molecule Real Time Sequencing (SMRT) [13] or Oxford Nanopore Technology’s minION [14] are already on the market. This is only the beginning – these technologies will mature and improve, and other ones are under development. This might eventually enable routine use of haplotype-resolved sequencing in clinical diagnostics and pharmaceutical research. So, in the next decade, when long reads become cheap and widely available, this will push to the forefront those methods that phase SNPs based on read information alone, the so-called *haplotype assembly* methods, a research area that has, until now, remained mostly of theoretical interest [15–17].

The haplotype assembly methods do exactly this: they assemble haplotypes from a set of sequencing reads. If two reads overlap on a SNP position, and their base-pairs at this position are different, i.e., they are *“conflicting”*, then one can deduce that they are on different alleles of the chromosome. The idea of this is that one can take this conflict information between pairs of reads to obtain a bipartitioning of the reads into two sets, i.e., the two alleles. This, combined with reads that link neighboring SNPs would give us a complete phasing of all SNPs, i.e., a set of haplotypes based on *direct observation*, in contrast to being based only on statistical information. This is where the long reads come in: they will someday provide this information, making haplotype assembly a much-needed tool for phasing.

Real data contains upstream errors, from the SNP calling phase, or the read-mapping phase, and so this becomes an optimisation problem: to obtain such a bipartitioning that involves correcting the minimum number of errors to the base-pairs of the reads. There are several different types of optimisation criteria in the literature, some of them equivalent. However we focus here on the *minimum error correction* (MEC) [18], as it is the most general of the criteria. Current read information is in the form of many short reads, that may pile up on certain SNP positions. Up to 2014, the current state-of-the-art of haplotype assembly methods [16, 17] solved MEC with approaches that scale, in terms of computational complexity, with the read-length. In addition to this drawback, these algorithms take advantage of the fact that many neighboring SNPs are not linked by these reads, because it allows to decompose this optimisation problem into independent subproblems. When reads get longer, these subproblems will no longer be independent – they should not be, since the goal is to link all of the SNPs. Also, a proportionally lesser *coverage*, i.e., the number of reads that cover a SNP position, will eventually be needed to obtain relevant information.

It is for these reasons that the authors of [19] introduced WHATSHAP, the first *fixed-parameter tractable* (FPT) [20] approach for solving the *weighed minimum error correction* (wMEC) [21] (and hence, the MEC problem) where coverage is the only parameter. The runtime of this approach is linear in the number of SNPs per read, which is the term that will increase by orders of magnitude as longer and longer reads become available.

A distinguishing feature of WHATSHAP with regards to the other currently available proposals is that it is exponential in the sequencing coverage and not in in read length. This appears to be very relevant when considering current trends in future generation sequencing technologies: technical improvements will clearly yield longer reads. The WHATSHAP algorithm has been implemented in a freely available toolkit (https://bitbucket.org/whatshap/whatshap).

Because WHATSHAP is the first approach in this promising direction, it appeared worthwhile to speed up its implementation by parallelising it. This paper presents PWHATSHAP, an optimised parallelisation of WHATSHAP, and its implementation in a toolkit which is also freely distributed (also available at https://bitbucket.org/whatshap/whatshap). PWHATSHAP has been a developing project, evolving together with the very active development of WHATSHAP. Preliminary results on the parallelisation experiment of the core structure of the algorithm were reported in [22]. In this paper we report on the parallelisation of the latest version of WHATSHAP, which has matured into an integrated framework engineered according to the current trends in genetic applications, and capable of analysing data in standard file formats (such as BAM and VCF) used in genomic analysis.

The merits of this work are:

- The pWhatsHap project provides the research community with a freely available application, which can easily be embedded in analysis pipelines requiring the solution of haplotyping problems. The core of the parallel haplotyping algorithm consists of an advanced and optimised implementation tailored to multi-core architectures. Such an enhanced core has now been engineered in the integrated framework described above, supporting standard data formats. This is a major engineering step, requiring the embedding of several C++ core functions, coherently running as a parallel application, into a framework developed in Python. This allowed the pWhatsHap project to move from a prototype development phase to a mature, open-source product. Haplotyping can be typically employed in larger pipelines, for instance including other typically expensive steps, such as data acquisition and result analysis. The provision of efficient solutions to haplotyping, such as pWhatsHap, empowers more accurate analysis in all those contexts.
- The incremental construction of haplotypes in WhatsHap is the type of algorithm whose parallelisation is very difficult. These algorithms process a large amount of data and are therefore sensitive to the availability of sufficiently large amounts of memory (RAM). Their exponential complexity (in time, but with direct implications on space complexity), and the huge datasets currently available, easily make memory availability a critical parameter. Parallelising one of the problems of this type represents an engineering challenge. The solution adopted is supported by the FastFlow framework [23], which provides high-level parallel programming constructs, such as *skeletons* and parallel design patterns. Thanks to the high-level programming paradigm adopted, it has been possible to build pWhatsHap retaining most of the overall structure and code of WhatsHap. The chosen paradigm has also the advantage to limit the need for mutual exclusion mechanisms, known to be typically slow. The clear performance improvement obtained supports the efficient treatment of large datasets and high coverage. It is important to note that the presented results can be obtained by computers that may easily equip current state-of-the-art genomic laboratories. Such improvement in the computational efficiency of haplotyping, made available at affordable costs, may be key in several analysis pipelines.
- A comprehensive evaluation of the obtained results has been carried out, both theoretically and experimentally. First, the *correctness* of pWhatsHap has been validated against WhatsHap: both applications return identical results in terms of the wMEC score of the computed optimal solutions. Following correctness, the *accuracy* of pWhatsHap has been discussed in terms of the accuracy of WhatsHap, which is known to be strong. We discuss various aspects of the accuracy of WhatsHap and review the several constraints under which the competing approaches to haplotyping work. pWhatsHap emerges as an accurate and largely applicable approach. The *efficiency* of pWhatsHap is discussed against theoretical complexity results, and validated by means of experimental results over benchmark datasets. Overall, the large applicability and accuracy of pWhatsHap, together with its increased efficiency, make it a reference player in the quick developing quest for solutions to the haplotyping problem.

In the next section, Methods, the problem of haplotyping will be defined and the WHATSHAP approach described. Then, the details of the construction of PWHATSHAP are illustrated and the choices made in the engineering of the parallel solution discussed. An account of FastFlow, the supporting high-level parallel programming framework, concludes the section. The Results section evaluates the performances of PWHATSHAP. Two main parameters are considered to illustrate the validity of the developed application: *accuracy* of the returned results, and *efficiency* of the computation. The accuracy of PWHATSHAP builds on top of the accuracy of WHATSHAP, as discussed. Efficiency, instead, is demonstrated by means of suitable experimental results on benchmark datasets. Concluding remarks follow.

## Methods

### Haplotyping: a fixed-parameter tractable solution to wMEC

The *haplotype assembly problem* takes as input a set of *reads* of a diploid genome that has been mapped to some reference genome. For such a genome, the SNP positions are known, and the set of alleles are arbitrarily re-labelled to 0 and 1 for each SNP position. This makes the input as a matrix with reads as rows and SNP positions as columns.

More formally, the input data for *n* reads and *m* SNP positions is organised in an *n*×*m* matrix *F*. The cells *f*_*i, j*_ of *F* have values in {0,1,−}, indicating whether the read *i* at SNP position *j* has the value of allele 0 or of allele 1, or it does not cover the SNP site at all, i.e., the respective read is *not active* at this SNP position. A *confidence value* (or *weight*) *v*_*i, j*_ is assigned to each active *f*_*i, j*_ as part of the input to the problem. The weight *v*_*i, j*_ is obtained at preprocessing as a combination of the confidence degree of that value after the sequencing phase (that is, the confidence of that specific base call) and after the mapping phase (that is, the confidence degree of having mapped that read at that SNP position). In this way, the weight gives a measure of how likely value of *f*_*i, j*_ is correct. That is, this weight represents the “cost” of flipping *f*_*i, j*_ in the optimisation problem wMEC, which aims to correct with higher priority the bases with higher probability of being inexact, as in [24].

We say that two reads *r*_*p*_ and *r*_*q*_ have a *conflict* at a SNP position *j* if they are both active and have different values at column *j*. If there were no errors, two reads in conflict necessarily come from different alleles. A *correct haplotype assembly* is a bipartitioning of the rows of matrix *F* (the reads) into a pair of *conflict-free* sets *R* and *S*. Both *R* and *S* contain each the whole set of reads that have been identified as belonging to the same haplotype. However, conflict-free bipartitioningss rarely can be found in existing datasets because of sequencing and mapping errors. Therefore, it is important to be able to determine a minimum-weight set of corrections to such errors capable of making the bipartitioning conflict-free. As an example, the following fragment matrix *F* has not a conflict-free bipartitioning of its fragments (*f*_1_,*f*_2_ and *f*_3_, one each row):

$$F\ =\ \left(\begin{array}{cc} 1_{9} & 1_{9} \\ 0_{3} & 1_{8} \\ - & 0_{8} \end{array} \right) $$

Subscripts are a measure of confidence of each datum, i.e., *v*_*i, j*_, the cost to be paid to “correct” it. The minimum cost, conflict-free bipartitioning *R*={*f*_1_,*f*_2_}, *S*={*f*_3_} can be obtained by correcting the element *f*_2,1_, i.e., flipping it to a 1 at a cost of 3.

Several heuristic proposal to solve the MEC, e.g. the greedy approaches of [25, 26] to assemble haplotypes of a genome, based on sampling a set of likely haplotypes under the MEC model [27], and the much-efficient follow-up, analogous to [28], and based on an iterative greedy approach to optimise the MAX-CUT of a suitably defined [29]. Improved perfomrmances do not impact on accuracy. Mousavi et al. [30] reduces MEC to MAX-SAT, which is then be solved by a heuristic solver.

A heuristic, by definition, provides no bound on the quality of the obtained results, what each of these above methods are. In order to solve optimally the MEC problem, several non-heuristic, exact algorithms exist in the literature. Examples include the integer linear programming techniques of [17, 31]. Another way to solve a problem optimally is fixed-parameter tractable (FPT) algorithms. Several FPT algorithms for the MEC have been developed in [19, 24, 32, 33]. Nonetheless, the complexity of [32] is exponential in the read length, or the number of SNPs per read, which will soon become larger quite quickly with the developments of sequencing techniques. In turn, HapCol ([33]) requires the fragment matrix to be gapless (that is, in a row of *F*, no ‘-’ can occur with 0’s and 1’s both on the left and on the right), which exclude the applicability to datasets with paired end reads. Also, given that the HapCol is exponential in the number of corrections (and in the coverage too, but less strongly that WHATSHAP), then it actually solves a constrained version of the MEC problem where the number of correction is bounded a priori. WHATSHAP [19, 24] is an algorithm that is fixed parameter tractable in the *coverage*, rather than in read length It is hence much more suitable to the trends in development of current sequencing techniques. Not long after the publication of WHATSHAP, a very similar algorithm that is based on belief propagation was developed independently by Kuleshov [34]. The following section gives a brief summary of the WHATSHAP algorithm.

### WHATSHAP: the algorithm

The original sequential WHATSHAP uses dynamic programming. It takes as its input the *fragment matrixF* (one row per *read*, one column per SNP position, and values in {0,1,−}) and a set of confidence values associated to the reads’ active positions. WHATSHAP returns a conflict-free bipartitioning of the set of reads of minimum cost, using a dynamic programming approach.

The *cost matrixC* built by WHATSHAP has the same number of columns as *F* (i.e., one column for each SNP), and is constructed in an incremental way, a single column at a time. *F*_*j*_ represents the set of active reads in the *j*-th column. *C*(*j*,(*R, S*)) is the cell in the *j*-th column of *C* corresponding to (*R, S*), one of the possible bipartitionings of *F*_*j*_. Then, WHATSHAP computes the minimum-cost *C*(*j*,(*R, S*)) of making (*R, S*) conflict-free, over all possible bipartitionings (*R, S*) of *F*_*j*_.

A read that spans several consecutive SNP positions induces dependencies across the columns, given that such a read must be consistently assigned to the same allele over all the positions for which it is active – e.g. read (row) 2 in the matrix of the previous example. When WHATSHAP computes the cost of the bipartitionings of *F*_*j*_ in order to construct the *j*-th column of *C*, the (minimum) cost that is inherited by constructing *compatible* partitionings in the previous position *F*_*j*−1_ must also be considered. Such a cost, on its turn, carries the price for consistency with the preceding columns.

In the initial column of *C*, which refers to (*R, S*)s belonging to *F*_1_, entries *C*(1,(*R, S*)) depend only on the cost of making *R* and *S* conflict-free (trivially no inheritance has to be considered here).

The cost $W(1)^{1}_{R}$ of making *R*⊆*F*_1_ conflict-free by flipping to 1s all 0s in *f*_*k*,1_ (for an *r*_*k*_∈*R*) is equivalent to the sum of the weights associated to the 0s which are flipped. Alternatively, we indicate with $W(1)^{0}_{R}$ the cost of making *R* conflict-free by flipping to 0 all the 1s. At any such step, WHATSHAP takes the alternative that is most advantageous:

$${}C(1,(R,S))\,=\, min\left\{W(1)^{1}_{R},W(1)^{0}_{R}\right\} + min\left\{W(1)^{1}_{S},W(1)^{0}_{S}\right\}\!. $$

When building column *j*-th, the cost associated to each partitioning is the sum of the cost coming from the column itself, computed as in the first column, and the cost of a compatible bipartitioning inherited from the previous column. That is, when computing *C*(*j*,(*R, S*)), with *j*>1 and (*R, S*) a bipartitioning of *F*_*j*_, the *local* contribution of the *j*th column is the minimum cost of making *R* and *S* conflict-free over the *j*th column of *F*. Then, the cost of ensuring that (*R, S*) is consistent on all the columns *i*<*j* must be added. This is the minimum cost of all the *C*(*j*−1,(*R*^′^,*S*^′^)), such that (*R*^′^,*S*^′^) is “compatible” with (*R, S*). A partitioning (*R, S*) defined at *j* and a partitioning (*R*^′^,*S*^′^) defined at *j*−1 are *compatible*, written (*R, S*)≅(*R*^′^,*S*^′^), when each element in *F*_*j*_∩*F*_*j*−1_, i.e., the active reads in both *j* and *j*−1, is assigned to the same subset in (*R, S*) and in (*R*^′^,*S*^′^). Importantly, in such incremental construction the cost in the preceding column *j*−1 summarises all correction costs made to keep (*R*^′^,*S*^′^) conflict-free from column 1 up to column *j*−1. It follows:

$$\begin{array}{@{}rcl@{}} C(j,(R,S)) & =\!& min\left\{\!W(j)^{1}_{R},W(j)^{0}_{R}\right\} + min\left\{W(j)^{1}_{S},W(j)^{0}_{S}\right\} \\ && + min\left\{ C(j-1,(R',S')) \ |\ (R',S')\cong (R,S)\right\} \end{array} $$

The implementation of such an algorithm for the *j*-th step consists of

1. All the possible (*R, S*) at *j* are defined;
2. Column *j* is made conflict-free and the minimum cost for this is determined;
3. The minimum-cost compatible partitionings computed at step/column *j*−1 are identified;
4. The entry *C*(*j*,(*R, S*)) is filled in with the sum of all outcomes of the previous two steps.

After the completion of the construction of *C*, the result of the input wMEC instance is contained in the conflict-free partitioning (*R*^∗^,*S*^∗^) of smallest cost in the final column. Such solution also encodes all the (minimum-cost) corrections made during the construction of *C*, based on assigning reads in *F* to partitionings compatible with (*R*^∗^,*S*^∗^).

The maximum number of bipartitionings computed in the construction of each column determines the complexity of WHATSHAP. At each column *j* the possible bipartitionings are $\phantom {\dot {i}\!}2^{|F_{j}|}$. Therefore, the complexity is exponential in the number of active reads at any position, i.e., the the *sequencing coverage* (see [19]).

The sequential version of WHATSHAP makes use of several optimisation to speed up the computation. Among them, one is actually relevant for its parallelisation: the order in which bipartitionings are taken into account. Specifically, when computing column *j* of *C*, the possible bipartitionings of *F*_*j*_ are processed in a specific order, that is, according to its *Gray code* ordering. Gray code guarantees that the binary representation of each bipartitioning differs from that of the previous one by only one bit, for example, 0001 and 0011 (here, as standard we assume that each bit represents the fact that an active read is assigned to either *R* or *S*). This entails that two subsequent partitionings differ only because of a *single* read moving from a set to another. This results in an incremental computation that is more efficient, since, the computation of the new cost for the subsequent partitioning comes from the cost of the previous one in constant time, because updating $W(j)^{1}_{R}, W(j)^{0}_{R}, W(j)^{1}_{S}, W(j)^{0}_{S}$ requires constant time when they differ only because of a specific single read. As we will see, this organisation is relevant when partitioning the workload in parallel tasks.

### WHATSHAP: an integrated toolkit for haplotyping

Since the first prototype described in [24], the sequential version of WHATSHAP is currently an integrated toolkit. To facilitate seamless integration into data analysis pipelines, a new command-line user interface supporting general file formats (BAM for alignments and VCF for phased/unphased variants) has been added. Considerable effort has also been invested into optimised algorithms for read pruning, e.g. in order to control the maximum coverage. Furthermore, the major modules have been reengineered in Python, a suitable and largely used development environment in Bioinformatics. The core haplotyping algorithm is still a C++ application.

### PWHATSHAP: high-performance WHATSHAP on multi-core architectures

The focus of this work is on parallelising the core haplotyping algorithm embedded in the WHATSHAP integrated toolkit described above. The main rationale behind such a choice are the desirable properties of WHATSHAP: solving wMEC with a complexity that does not depend on read length, but is exponential only *in the sequencing coverage*. This appeared to be particularly relevant when considering the future trend of sequencing technology, which are inching towards longer reads. Furthermore, solving the weighted version of the problem caters to its accuracy.

Two main approaches to parallelisation can be followed, respectively focusing on the haplotyping of a *single* chromosome or *many* of them. Actually, single chromosome datasets that can be decomposed in “independent” sets of SNPs, i.e., no read covers any two of these sets, can be addressed as if the sets were belonging to different chromosomes. The many instances of haplotype assembly required for the different genes of a whole genome, or independent sets of SNPs of the same gene, are completely independent. They can be run concurrently in an *embarrassingly parallel* fashion. Since haplotyping is a memory-bound algorithm, it exhibits the best scalability when executed on distributed platforms (e.g. clusters or cloud resources) where the memory hierarchy and the file system are not shared resources among executors. Independent runs of PWHATSHAP could be supported by the cloud computing services, which are regarded as enabling technologies for bioinformatics and computational biology because they can provide pipelines with computing power and storage in an elastic and on-demand fashion. In this paper we address the parallelisation of the core haplotyping algorithm for a *single* chromosome, and the consequent development of the PWHATSHAP toolkit, i.e., the parallel version of the WHATSHAP toolkit. In this, we directly selected multi-core as target platforms class for three fundamental reasons: 1) simplicity of porting; 2) minimal disruption with respect to existing sequential code; 3) concurrency grain availability in the fine- to very fine-grained range.

### PWHATSHAP: the parallel algorithm

In the parallelisation of the core haplotyping algorithm for a single chromosome, the structure of WHATSHAP clearly imposes strong constraints on the parallelising approach that can be followed. The incremental approach of WHATSHAP when building the solution, i.e., the column-wise exploration of the input matrix, imposes a strong linear dependency of each step on the immediately preceding one. This makes very difficult to imagine a possible decomposition of the problem by sets of columns that can be independently processed in parallel.

Given that WHATSHAP follows the described linear incremental construction of a solution by columns, and this makes the decomposition of the problem in sets of columns independently processed not viable, a “row-based” parallelisation has been adopted. Each parallel executor processes a number of the elements (rows) of the column of the cost matrix under consideration, that is, each executor evaluates some of the bipartitionings (*R, S*) of *F*_*j*_, which are the active reads on column *j*. A column-based decomposition, as well as hybrid solutions possibly mixing the two approaches, are the scope of future work.

The first step when moving from the sequential design of WHATSHAP to a row-based parallel implementation was *profiling* the efficiency of WHATSHAP in terms of the *time* needed to generate the *j*-th column of *C*, the minimum cost matrix *C* (see p. 5). This is useful to determine whether a column of a given coverage requires enough work to be worth parallelising it. Table 1 shows data from a profiling test on a given dataset. The time required by the sequential algorithm for processing a column is reported in the second row, according to the column dimension. This is a function of the number of possible bipartitionings of the active reads on the column, i.e., it depends on the *coverage* (there are ∼ 2^*c*^ possible bipartitionings for coverage *c*). It is easy to appreciate its exponential growth. From the results summarised in the table, the cost for the smaller columns (coverage <15) is negligible, less than one *ms*, therefore not justifying the parallel overhead. Differently, when *c*>15, the cost varies from a few milliseconds to a few seconds for each column (for *c*>25). Columns with coverages bigger than 16/18 are worth being parallelised.

**Table 1:** WHATSHAP profiling. Test for an input data sample with coverage 20 on a 2 CPU Xeon E5-2695 @2.4 GHz, 12-core x 2 context for each CPU, 64 Gb RAM

Coverage	<15	15	16	18	20	22	24	26	28	30
Time (ms)	<1	1.1	2.2	8.7	34.2	144.7	558.5	2352.7	9194.3	36622.7

What is also interesting is to gauge how many columns worth being parallelised are present in a given dataset. This of course is highly dependent on the specific dataset, but carried out experiments show that a sufficiently large number of high-coverage columns justify the parallelisation, as shown in the section Results. Statistical analysis of this kind are useful to predict the gain that can be achieved. Depending on factors like the specific architecture, the incurred overhead of parallel executions, and data distribution, it might be worth it to implement an adaptive partitioning, where the number of executors is tuned to the dimension of each column. After some empirical validation, we have abandoned this possibility because it did not appear to be of much value for our reference architecture and settings. Overall, this appears as a fine-grained algorithm, typically difficult to be parallelised, but, interestingly and not surprisingly, the best speed-ups can be obtained with large coverages, which are of great interest, since they provide increased accuracy.

**Fig. 1:**
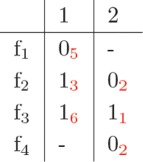
The fragment matrix *F*

In the following, the parallel construction of a minimum cost matrix *C* that we designed for PWHATSHAP is presented through a simple example (an elaboration of the example firstly introduced in [22]). Let us consider the fragment matrix *F* in Fig. 1, which has two columns only, with associated weights (in red). In *F*, for instance, read *f*_1_ is 0 in SNP 1 with confidence 5, while read *f*_2_ covers SNPs 1 and 2, where is 1 and 0 with confidence 3 and 2, respectively.

**Fig. 2:**
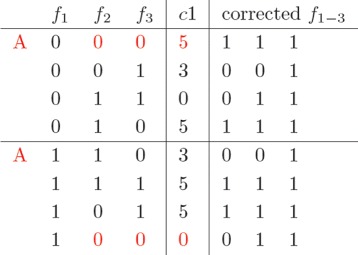
The cost matrix *C*(1,(*R, S*))

The cost matrix *C*(1,(*R, S*)), reported in Fig. 2 and associated to the first column of *F*, is built by considering all the possible bipartitionings (*R, S*) of the reads active on SNP 1, i.e., *f*_1_,*f*_2_ and *f*_3_. In the matrix *C*(1,(*R, S*)), partitionings are represented as binary strings in Gray-code order (see p. 5), as reported in the first three columns. In the example under consideration, the set of all possible bipartitionings is split between two executors (horizontal line). Parallel executors work on disjoint section of the partitioning space. In order to retain as much as possible the original structure of the sequential algorithm, bipartitioningss are processed sequentially by each executor according to the Gray code order. A bit of care is necessary to properly identify the entry points for each executor, i.e., the *A*s in red in the matrix, in the Gray code sequence. Suppose that an executor is expected to process a set of partitionings starting from the *r*−*th* one. This will not necessarily be identified by the *r*−*th* binary number, as expected, but actually by the *r*−*th* entry in the Gray code. For instance, in the matrix, the second entry point *A* is not 100, as one would expect, but 110.

Each entry in the column *c*1 in the matrix *C*(1,(*R, S*)) reports the cost of making the corresponding partitionings conflict-free. This is the only cost incurred so far, dealing with the construction of the first column. For instance, partitioning ({*f*_1_,*f*_2_,*f*_3_},*∅*) (first row) requires flipping *f*_1_ to 1 at a cost of 5 (column *c*1), so that *R* is conflict-free and *S* empty.

The last three columns of *C*(1,(*R, S*)) show the corrected values of the reads.

**Fig. 3:**
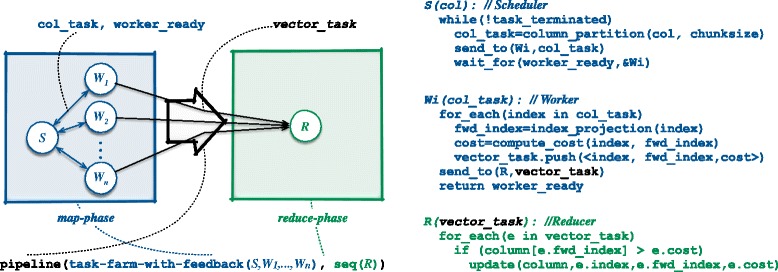
The FastFlow skeleton used in PWHATSHAP. Each entity is a concurrent thread. The Emitter (*S*) produces and schedules tasks towards a pool of Workers (*W*s). Each Worker sends results to the Reducer (*R*) and asks for new tasks from *S*

Considering *C*_*j*_, the *j*-th column of *C* and *k* executors, each executor computes a number of bipartitionings of *F*_*j*_ in the range of $\phantom {\dot {i}\!}2^{c_{j}}/k$, with *c*_*j*_ the coverage and *k* that may be dynamically adapted according to the coverage (and the current hardware features). Each one of the *k* executors processes the assigned bipartitioning in parallel. This is the *map-phase*, see Fig. 3.

**Fig. 4:**
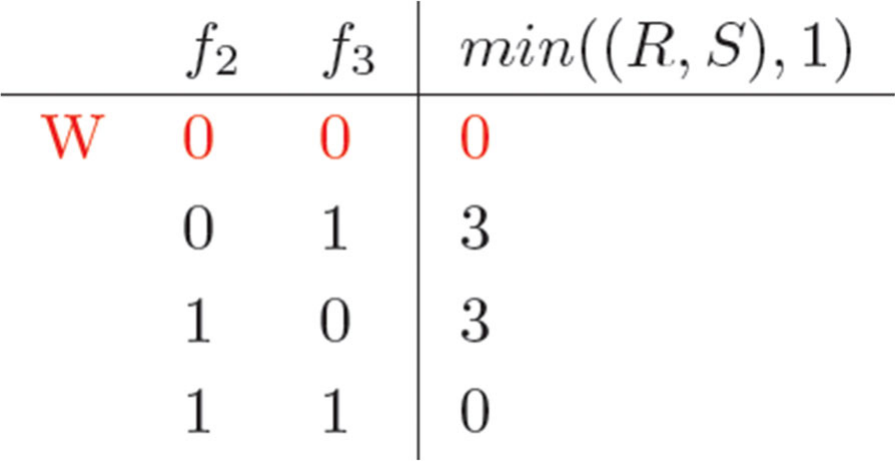
Cost information about compatible partitionings between any two columns

In the construction of *C*_*j*_, the cost of any specific bipartitioning of the reads active on the *j*−*th* column depends on the *minimum* costs of the bipartitionings in *C*_*j*−1_ which are *compatible* with that partitioning. In our example, *f*_2_ and *f*_3_ are active in both columns 1 and 2. Bipartitionings ({*f*_1_,*f*_2_,*f*_3_},*∅*)_1_ and ({*f*_2_,*f*_3_},{*f*_4_})_2_, from columns 1 and 2 respectivley, are compatible and could eventually lead to ({*f*_1_,*f*_2_,*f*_3_},{*f*_4_}). Instead, ({*f*_1_,*f*_2_,*f*_3_},*∅*)_1_ and ({*f*_2_},{*f*_3_,*f*_4_})_2_ are not compatible (see p. 5). Cost information about compatible partitionings between any two columns is recorded in a suitable matrix (Gray code ordered). In our example, such matrix would be the one reported in Fig. 4: each executor *over-writes* the currently discovered best cost for that specific partitioning. This may cause write conflicts, whenever different executors report costs associated to the same row. In the example this is indicated by *W*, in red, in the matrix, and it is due to the two executors working on *C*(1,(*R, S*)) and attempting to update the (minimum) cost of having both *f*_2_ and *f*_3_ in the “0” partitioning. Note that there are two cases in which this happens, marked in red in the partitioning columns of *C*(1,(*R, S*)) in Fig. 2 (first and last row), and these two cases are being dealt with by different executors. Such case of write conflict has been addressed by constructing local copies of the table for each executor, and then managing their merging by means of a sequential *reduce-phase*, executed in pipeline with the *map-phase* (Fig. 3).

**Fig. 5:**
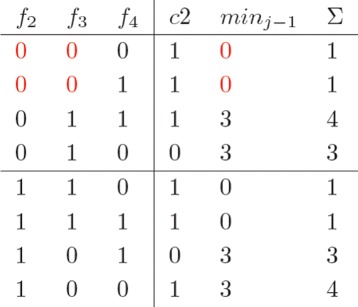
The cost matrix *C*(2,(*R, S*))

Minimum costs recorded as in Fig. 4 are then accumulated in the definition of the so-far-incurred costs in the construction of the cost matrix for the next column of the fragment matrix *F*, as shown in Fig. 5 (corrected values of fragments omitted). This last matrix is built on top of the three reads in the second column of *F*. The *c*2 column reports the cost of the local corrections for making each partitioning of {*f*_2_,*f*_3_,*f*_4_} conflict free, as standard. The *min*_*j*−*i*_ carries over the minimum costs recorded in the previous table (column *min*((*R, S*),1) in our example). The last column *Σ* reports the so-far-incurred minimum costs to make each partitioning conflict-free as the sum of the previous two columns. Possible concurrent *read* accesses to the previous table, as the ones in red (the 0 in *min*((*R, S*),1) is copied twice - possibly by different executors, in *min*_*j*−1_), are of no particular concern.

The partitioning ({*f*_1_},{*f*_2_,*f*_3_,*f*_4_}) is *conflict free* and *minimal cost*, once that *f*_3_ has been corrected in [1,0] at the cost of 1. This is an optimal solution found by PWHATSHAP, built in the last and first rows, respectively, of the two cost matrices above.

It is worth remarking that whenever two or more solutions with the same minimum cost exist, due to the interplay of the different amounts of time spent by different executors to accomplish their parallel tasks, non-determinism may occur when overwriting minimum costs, and, as a consequence, different optimal solutions of same cost can be returned from different runs. The comparison and properties of such equivalent solutions is scope for future work.

### PWHATSHAP: the parallel implementation

The focus of the present work is the parallelisation of the core WHATSHAP haplotyping algorithm, which is a component of a larger application whose main module is written in Python, with Cython used for interfacing Python and C++. Our starting point is the WHATSHAP core algorithm written in C++, which is actually embedded into a larger, multi-language application, making the development of the parallel version very elaborated, for instance requiring us to work on the edge of different programming paradigms during both debugging and tuning.

The parallel construction of the minimum cost matrix *C* proceeds independently over the possible bipartitionings (*R, S*) of the current column *F*_*j*_. We aimed to exploit the maximum possible parallelism in this construction by exploiting both task and data parallelism. For this we used a pipelined map-reduce paradigm, i.e., *pipeline(map-phase, reduce-phase)*.

In the *map-phase*, all the possible bipartitionings of the fragments in *F*_*j*_ are generated; their cost is also computed. In the *reduce-phase*, the cost matrix *C* is updated with with the minimum cost found among all the bipartitionings generated in the previous stage.

In FastFlow, this can be easily realised by nesting patterns implementing map and reduce phases within the pipeline pattern. The *map-phase* can be implemented by way of the task-farm-with-feedback pattern, which make it possible to execute independent tasks in parallel, i.e., generate and analyse all the possible bipartitionings. The *feedback* loop feature enables the pattern to implement a effective dynamic load balancing strategy. The *reduce-phase* can be implemented in a single worker since it is much lighter than the *map-phase* and is actually never a bottleneck for the whole process. Overall:

pipeline(task-farm-with-feedback(*S, W*_1_,…,*W*_*n*_), sequential(*R*))

where *S* is a task scheduling, *W*_*i*_, *i*=1..*n* is array of workers for the *map-phase*, and *R* is a reducer worker for the *reduce-phase* (see Fig. 3. In the *map-phase*, the *S* thread, by using a dynamic scheduling policy, sends tasks having a computation granularity proportional to *chunksize* towards the workers *W*_*i*_. Each worker *W*_*i*_, stores results in a local data array (thus avoiding the need of mutual exclusion for accessing global data) and eventually sends the produced data as a single task to the second stage of the overall pipeline (*R*). This way, for each worker’s input task, is produced an output task containing maximum *chunksize* different results. The second stage receives *tasks* from all workers (i.e., locally produced results) and then updates the cost matrix *C* with the minimum cost found (*reduction* phase on all inputs received). The *R* thread, is the only thread that performs write accesses to the cost matrix.

Overall, it is possible to exploit: 1) Scheduler–Workers pipeline parallelism: the scheduler *S* computes all possible bipartitionings sending disjoint sub-partitionings to Workers *W*_*i*_ using a dynamic scheduling policy; 2) parallelism among Workers: the computation of local minimum costs proceeds in parallel in all the *W*_*i*_; and 3) Workers-Reducer pipeline parallelisms: the Reducer *R* receives multiple results in chunks from each worker.

It is worth noting that, the parallelisation strategy just described, is applied to only those columns that have a coverage larger than a given size (the *THRESHOLD* value).

This is because, the overhead introduced in the parallelisation of an excessively fine level of granularity with respect to computation (due to synchronisation among threads and to the creation of extra data structures), might overcome the advantages of the parallel execution. For this, is necessary to cut the application of parallel computing to kernels exploiting a minimum level of granularity. As we shall discuss in the Results section, for PWHATSHAP the threshold value is set around coverage 20, this value being almost independent of the input dataset considered.

The proposed parallelisation is quite direct and, importantly, requires minimal changes to the original sequential WHATSHAP code. Furthermore, a high degree of parallelisation is involved due to the many entries of the large *fragment tableF* corresponding to many (small) tasks that can be executed in parallel on the available cores.

### The FastFlow parallel framework

FastFlow [23] is a programming framework supporting high-level parallel programming for shared memory multi/many-core and heterogeneous distributed systems. It is implemented in C++ on top of the Posix Threads and the libfabric standard interfaces and provides developers with a number of efficient, high-level parallel programming patterns.

The framework offers a methodological approach that allows applications to be rapidly and efficiently parallelised on a broad range of multi/many-core platforms. Thanks to its efficient lock-free run-time support [35], applications developed on top of FastFlow typically exhibit a good speedup because of the reduced synchronisation cost (about 20–40 clock cycles) and with a minimal tuning effort.

The parallelisation of WHATSHAP here presented is based on FastFlow. It exploits the cache-coherent shared memory of the underlying architecture, making it unnecessary to move data between threads, which is a typical source of overhead. However, if shared memory greatly simplifies the parallelisation, it also introduces concurrent data access problems which eventually turn into synchronisation overheads. Parallel patterns defined and implemented by the FastFlow framework solve these problems by defining clear dependencies among different parts of the computations, hence avoiding costly synchronisations.

FastFlow has proven to be effective in parallelising a broad class of sequential applications and in redesigning concurrent applications originally developed with low-level abstraction programming tools, which typically hinder portability and performance on new multi-core platforms, e.g. [36–38]. For the development of parallel version of WHATSHAP, FastFlow offered a methodological approach capable to support the parallelisation while keeping the needed modifications to the sequential code at a minimum.

## Results and discussion

The PWHATSHAP project focused on the design and development of a high-performance, parallel application for the solution of the haplotype problem. This has been done building upon the WHATSHAP framework, an evolving tool-kit which currently supports several stages in the haplotyping pipeline and supports data analysis in standard formats. As illustrated, the choice of WHATSHAP is justified by its performance in terms of *accuracy*, i.e., being able to provide solutions with a low percentage of errors, and its interesting *computational complexity*, which depends on the coverage of data sets rather than on the length of reads. This appeared as a desirable property in the light of the future trends in sequencing technologies that will yield longer and longer reads. Indeed, other proposals based on similar approaches to computational complexity are being developed.

Building upon the feasibility study presented in [22], PWHATSHAP addresses in particular the efficiency of the core algorithm for the construction of correct haplotypes, and provides a multi-core, high-performance version of it that is fully integrated with the other stages of the WHATSHAP framework. Thanks to the parallelisation technique adopted, which requires minimal modifications to the the sequential code, the developed solution retains the accuracy properties of WHATSHAP.

A detailed description of the *accuracy* and *efficiency* properties of PWHATSHAP is reported in the following. Accuracy reduces to the accuracy of WHATSHAP, since the sequential and parallel frameworks return identical results in terms of the wMEC score, i.e., solutions of the same minimal cost, although PWHATSHAP can return a richer set of cost-equivalent solutions than WHATSHAP. Therefore, the accuracy of PWHATSHAP can be properly accounted for on the basis of the results existing in literature on the accuracy of WHATSHAP. Efficiency instead has been validated by suitable tests on a medium-size, shared-memory, multi-core computer, which could reasonably equip a genomics analysis facility. Test results show the effectiveness of the parallel PWHATSHAP developed, as far as the core haplotyping module is concerned.

### Accuracy

In this section we compare the accuracy of PWHATSHAP against the accuracy of state of the art approaches to haplotyping. As explained, this is done by exploiting existing data about the accuracy of WHATSHAP, given that PWHATSHAP exhibits the same behaviour as WHATSHAP. In order to make this explicit, we will use (P)WHATSHAP where appropriate in the rest of this section.

The accuracy of reconstructed haplotypes can be validated by considering both *error rate* [39], that is the count of phased variants presenting some discrepancies, and *phased positions*, that is the count of genomic positions for which a phased prediction can be identified out of all the phasable positions in the whole dataset. (P)WHATSHAP is compared to three tools which have been specifically designed for the long reads coming from third generation sequencing technologies: ProbHap [40], a recent approach that uses a probabilistic graphical model to exactly optimise a specific likelihood function; RefHap [41], a heuristic method presenting very high accuracy; and HapCol [33], a Fixed-Parameter Tractable algorithm that computes linearly in relation of the number of SNPs and exponentially in function of the coverage. More precisely, HapCol’s time complexity is in $O\left (\sum _{s=0}^{k}{cov \choose s} \cdot cov \cdot L \cdot m\right)$, where *L* is the length of the read, *m* the number of SNPs, *cov* the coverage, and *k* is HapCol’s input parameter of the maximum number of errors it corrects per column, while WHATSHAP’s complexity is in *O*(*m*·2^*cov*−1^).

Both a real and a synthetic data set have been considered for the comparison. The real dataset (the sample NA12878) was analysed in the HapMap project [41] and it is a standard benchmark for haplotyping algorithms designed to work with long reads, since the haplotype of this patient, and also those of her parents, was independently reconstructed using genome sequencing techniques. The dataset consists of 271,184 reads with average length of ∼40 kb and with average coverage of ∼3x. Variant calls have been achieved using the GATK [42] considering the 1,252,769 positions covered by the NA12878 dataset and are trio-phased. (P)WHATSHAP, RefHap, HapCol, and ProbHap have been tested on each chromosome independently. The dataset used does not include paired end reads because HapCol cannot handle them. Moreover, despite the fact that (P)WHATSHAP and HapCol can compute haplotypes outside the all-heterozygous hypothesis (which allows for a better handling of sequencing errors, since it permits to consider a SNP site homozygous also if its column is non-monotone), in this test case, the all-heterozygous assumption was enforced for all the tools. Even if the all-heterozygous assumption has no impact on their time/space complexities, the comparison between solutions achieved under different hypothesis may produce misleading results. Considering that all the SNPs in the dataset are heterozygous with high confidence, this assumption is not strictly necessary in this case.

**Fig. 6:**
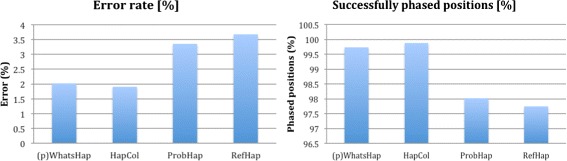
Accuracy comparison amongst state of the art toolkits. (P)WHATSHAP (first-left in the histograms) is top in minimising errors as well as in properly phasing, together with HapCol. Data extracted from [33]

Figure 6, built from data in [33], shows, for the different tools, the error rate (left histogram) and the percentage of phased positions compared to the total number of positions which can be phased in the input reads (right histogram). Considering this dataset, both HapCol and (P)WHATSHAP achieved very good results in terms of accuracy, reconstructing the haplotypes with high precision and phasing a large number of positions compared with the other two tools. In particular, HapCol and (P)WHATSHAP improved the accuracy of the other two tools by more than 40 %. Incidentally, WHATSHAP also performed fast, 172 s, behind RefHap, 43 s, and ahead of HapCol, 332 s, and ProbHap, 1205 s.

In [33] a synthetic dataset was also generated and used for comparative analysis on accuracy. Specifically, the analysis aimed to assess how accuracy changes while varying the coverage of the dataset. Given that the real, standard benchmark dataset previously used relies on the all-heterozygous assumption, and hence contains only heterozygous SNP positions and has low average coverage, a synthetic datasets has been used to characterise the behaviour of tools against the long reads that will be soon available thanks to future-generation sequencing technologies (max coverage 25 ×, max read length 50,000 bases, max indel rate 10 %, max substitution rate 5 %).

The dataset has been generated inserting all the variants of chromosomes 1 and 15 of the Venter’s genome into the hg18 assembly genome. Long reads have been generated at length 1000, 5000, 10,000, and 50,000 using a uniform indel distribution of 10 % and substitution rates 1 and 5 %. These rates have been defined according to the information currently available about the accuracy of long read data generated using future-generation sequencing technologies (see, e.g., [43, 44]). The final in silico datasets were achieved extracting from each set of simulated reads subsets with maximum coverage of 15 ×, 20 ×, and 25 ×.

**Fig. 7:**
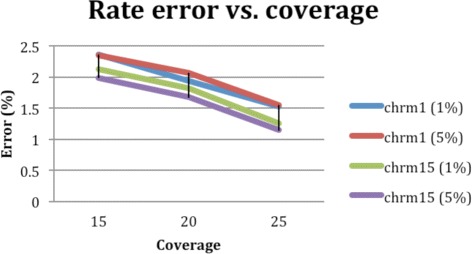
Accuracy as error rate for increasing coverages. The curves in figure show how the accuracy scales up (error rate decreases) with larger coverages. Curves represent data for Venter’s Chromosome 1 and 15 with substitution error rate 1 and 5 %. From coverage 15 to coverage 25 the error rate decreases by about 40 %. Based on data extracted from [33]

Since ProbHap and RefHap require that haplotypes are computed outside the all-heterozygous hypothesis, only tests regarding (P)WHATSHAP and HapCol are relevant. Data in [33] shows a substantial coherence of (P)WHATSHAP and HapCol in terms of accuracy (less than 1 % of differences), and illustrate how accuracy, measured as error rate, improves with larger coverages. Trends of such improvements are reported in Fig. 7. Such data provides further grounds to the interest for PWHATSHAP, whose speed-up increases with coverage.

Although HapCol, together with WHATSHAP, has high accuracy on these datasets, it is worth recalling that HapCol has a couple of substantial drawbacks with respect to WHATSHAP. The first one is the above mentioned requirement for the fragment matrix *F* to be gapless, which results in the heavy limitation of not being usable with paired end reads. The second one is that HapCol actually solves a constrained version of the MEC problem (which is called *k*−*cMEC* in [33]) that limits to a given parameter *k* the amount of errors that can be corrected. This is due to efficiency reasons, because HapCol takes time and space exponential in the amount of corrected errors. Moreover, for the same reason, HapCol actually requires the assumption that errors are uniformly distributed, which is not very realistic for certain sequencing technologies. Finally, the computational complexity of HapCol is also exponential in the coverage, even if not as strongly as WHATSHAP.

### Efficiency

In this section we outline results of experiments aiming at assessing the performance of the proposed parallel algorithm. All the experiments have been performed on a platform equipped with two E5-2695@2.40 G Hz Ivy Bridge Intel Xeon processors, each with 12 cores, 64 G Bytes of memory, Linux Red Hat 4.4.7 with kernel 2.6.32. CPU dynamic frequency scaling and turbo frequency boost have has been disabled to ensure a fair comparison among codes using a different number of cores. Both parallel and sequential codes have been compiled with gcc 4.8.2 using –O3 optimisation level. The parallel version was executed using the shell command numactl –interleave=all to exploit all the available memory bandwidth of the 2 NUMA nodes of the hardware platform.

Experiments have been run on a range of synthetic data sets with maximum coverage of 16,18,20,22,24,26 and 28, which have been generated from a single data set with an average coverage of 30, mapped to human genome and then pruned to smaller coverage data sets (see [24] for details on the construction). Such coverages correspond to fairly large data sets. Performance has been evaluated by measuring the computation time elapsed in the computation of subsets (i.e., a given number of columns) of each data set. The dimension of each subset was chosen to guarantee that the entire produced output could fit in main memory.

Firstly, we executed a set of tests aimed at assessing the time needed to compute columns of different coverage. On the considered platform we observed that it is worth parallelising only columns with a coverage ≥20; we define them as *higher coverage* columns. Columns with coverage of 20 have an average computation time of about 35.7 ms. The average time from processing columns with a coverage <20 is less than 10 ms; we defined them as *lower coverage* columns.

For higher coverage columns, we observed that the best execution time was obtained by using all the cores of the platform (24), specifically 23 worker threads for the map phase and 1 thread for the reduction phase. Conversely, for columns with lower coverage the synchronisation overhead exceeds the performance gain, thus they are computed with sequential code.

The speedup of the proposed parallel PWHATSHAP against the original sequential WHATSHAP is reported in Table 2. Specifically, the table reports the average computing time for a column for the reference dataset, filtered by different maximum coverages. For each filtering, the WHATSHAP and PWHATSHAP performance is reported together with the speedup of PWHATSHAP over WHATSHAP, defined as *Speedup = TSeq/TPar*. For all coverages, the amount of main memory used was fixed to ∼63 GB in all the tested cases.

**Table 2:** Overall speedup considered for the dataset filtered for different maximum coverage figures

max cov.	Avg. time/col. (ms)		Speedup
	*TSeq*	*TPar*	
16	0.3	0.3	1.0
18	0.6	0.6	1.0
20	2.4	2.3	1.1
22	11.1	5.2	2.1
24	47.4	14.3	3.3
26	180.9	44.7	4.0
28	1462.5	287.9	5.0

Considering the case of the dataset filtered for max coverage 28, the fraction of sequential time, including both the columns whose construction is not parallelised and inherently sequential parts of the application, amounts to about 15.6 *%* of the overall computation time. In that case, from Amdahl’s law [45] it follows that the maximum possible speedup would be around 6.4. Indeed, if *f* is the fraction of the algorithm that is strictly sequential, i.e., 15.6 *%* in our case, which is about 1/6.4, then the theoretical maximum speedup that can be obtained with *n* threads is $S(n)=~1/\left (f+\frac {1}{n}(1-f)\right)$, i.e., 1/*f*≃6.4 with *n*→*∞*.

The average execution time for computing columns with fixed coverage for several different coverages is reported in Table 3. The per-column gain obtained, is in the range 1–5.3, with a gentle but monotonic increase of speedup in the tested range. Due to the rapid increase of used memory, the biggest coverages in the table are somehow limit cases for speedup increase, since memory limitations strongly affect performances. As previously discussed, due to Amhdal’s law, a further significant increase of speedup will probably require improvements in the non-parallel parts of the algorithms, possibly leading to a major restructuring of the code.

**Table 3:** Speedup on columns with a specific coverage and % of dataset with the given coverage. Dataset is filtered for max coverage 28

col. cov.	% of dataset	Avg. time/col. (ms)		Speedup
		*TSeq*	*TPar*	
16	2.0 %	2.3	2.3	1.0
18	2.4 %	9.0	9.0	1.0
20	2.5 %	35.7	32.8	1.1
22	3.6 %	153.1	41.4	3.6
24	3.2 %	557.1	139.6	3.9
26	2.8 %	2461.0	585.3	4.2
28	12.0 %	9555.5	1175.5	5.3

## Conclusions

The work presented in this paper contributes to the haplotype assembly approach, a developing methodology for phasing SNPs based on direct evidence from reads obtained by DNA sequencing. Phasing grants us a better understanding of haplotype information, which is relevant in many contexts, including gene regulation, epigenetic, genome-wide association studies, evolutionary selection, population structure and mutation origin.

In this context, our contribution consists of a framework, PWHATSHAP, that improves the efficiency of state of the art haplotype computational analysis. Importantly, PWHATSHAP is aligned with the future trends of sequencing technology, which will provide long reads, i.e., long fragments of DNA sequences. Building on WHATSHAP, PWHATSHAP improves the efficiency of solving the weighted MEC optimisation problem for haplotyping and supports a faster analysis of datasets with large coverage. This also caters to the accuracy of the results, which in the current settings, increases with coverage.

PWHATSHAP is a multi-core, parallel porting of WHATSHAP. Experimental results and benchmark tests show increased performance that can be obtained using computational facilities which are available today at affordable costs. The core haplotyping algorithm is embedded in a larger framework, the same as WHATSHAP, which enables the treatment of standard formats for sequencing datasets. As PWHATSHAP is distributed as a freely available toolkit, our contribution aims to be widely accessible to researchers, as well as companies.

The development of PWHATSHAP has been a challenging parallelisation exercise for a fine-grained, data intensive algorithm. Such features made the process difficult. We have addressed this by exploiting FastFlow, a high-level parallel programming framework specifically targeting the parallelisation of fine-grained tasks, which allowed us to develop PWHATSHAP with minimal modifications to the sequential code.

Common to similar frameworks dealing with large datasets, a critical aspect of PWHATSHAP is the trade-off between memory usage and performance. A large amount of information is currently kept in memory for efficient access. However, the amount of available memory represents a rigid limit, after which the necessary virtual memory management and swap to secondary memory devices, i.e., disks, start to have an impact on performance. We envision two possible approaches to solve this problem and push even further the efficiency of PWHATSHAP.

The first one is based on optimised, ad-hoc memory management. The memory access pattern is fully sequential: a large bulk of data is sequentially written, then sequentially read in reverse order to build the solution. Data is never accessed in random order except for the very last column. An intelligent memory management, aware of such problem-specific information, could maintain relevant data in a limited amount of memory while needed, and swap to disk data outside such a working set (i.e., almost all but the last two columns). The difficulty lies in providing programmers with suitable abstractions that allow them to transparently deal with data swapping, i.e., technically, a user-space virtual memory optimised to manage the sequential data scheme used by PWHATSHAP.

The second approach is based on memory compression, which is making a comeback mainly because of the availability of multiple core processors. Memory compression has been considered recently in projects regarding Linux, ChromeOS, Android and OS X. Intelligent memory compression would also exploit haplotyping specific information. The two approaches could be combined together, and paired with advanced data management techniques.

The large availability of cores would allow such data management processes to be offloaded to one or more processor cores in a quite seamless way.

This is the scope of future developments.
